# Association between community environment and dependency among the elderly people from a service provision perspective

**DOI:** 10.1186/s12877-022-03687-z

**Published:** 2022-12-13

**Authors:** YiYang Pan, Yuan Chen, PingYu Cui, Nuremaguli Waili, Ying Li

**Affiliations:** Department of Social Medicine, School of Public Health, Zhejiang University, 866 Yu-Hang-Tang Road, Hangzhou, 310058 Zhejiang China

**Keywords:** Dependency, Place, Community environment, Resource dependency, Elderly people

## Abstract

**Background:**

The incidence of dependency is high among the elderly people worldwide and increases with increasing life expectancy. The purpose of this study was to establish from the perspective of resource demand the association between community environmental resources and dependency among the elderly people.

**Methods:**

This study is a cross-sectional design based on community from 22 locations in China. A multistage sampling method was used to select the study objects. The questionnaires were used to collect the survey data by face-to-face interviews. A total of 950 individuals completed the survey, and 913 individuals were available for this analysis. Dependency and community environment were measured using the standard instruments. Logistic regression analysis was performed to identify the community environment factors associated with dependency. Cluster analysis was used and demonstrated that dependency was mainly associated with community primary preventive care service resources.

**Results:**

In the group aged under 70 years, the utilization of electronic health records and the need for health assessments, and rehabilitation equipment rentals were significantly associated with the levels of dependency scores: the OR was 2.81, 2.25 and 2.13 (*P* < 0.05), respectively. In the group aged 70 years and over, a short-term care home was strongly associated with levels of dependency: the OR was 4.01 (*P* = 0.002). The daycare and nursing service, transportation service, and regular lectures on health knowledge were associated with levels of dependency: the OR was 2.41, 1.86 and 1.93 (*P* < 0.05). In the group with low social support, an emergency call or monitoring system, transportation services, the need for health assessment, and regular lectures on health knowledge were significantly associated with levels of dependency: the OR was 2.42, 2.19, 1.89 and 1.98 (*P* < 0.05), respectively.

**Conclusions:**

Community environment resources were significantly associated with dependency. These results suggest that the dependent on local environment resources may consider as the resource needs among elderly people.

**Supplementary Information:**

The online version contains supplementary material available at 10.1186/s12877-022-03687-z.

## Background

Owing to the accelerating aging of the global population, the realization of healthy active aging has become a common concern worldwide [1, 2]. As the elderly people age and their physical function declines, the increasing prevalence of their dependency leads to increasing demand for medical care and social services [3, 4], substantially challenging the individual and social service system and becoming an urgent problem requiring immediate solutions [5].

Dependency is defined as a personality disorder in Diagnostic and Statistical Manual of Mental Disorders. Individuals are heavily dependent on others that lead to the loss of initiative and autonomy. [6, 7]. The causes of dependency are not very clear, and its classification and consequences are complex. A recent study demonstrated that markers of biological age, such as leukocyte telomere length, are associated with dependency [8], but health and the consequences of poor health and dependency are more intimately linked with social, economic, and environmental factors, especially for the elderly people [9, 10]. Many studies have demonstrated the adverse outcomes caused by different dependency objects such as people, substances, and behaviors, which can lead to nursing dependency [11], alcohol dependency [12], and sleep dependency [13]. Dependency can also lead to increases in the consequences of diseases such as depression, heart disease, and all-cause mortality [14].

The theory of place dependency [15] was proposed more than 30 years ago, but quantitative studies on the association between person and place dependency have rarely been conducted [16, 17]. Because the performance of dependency is multifaceted, and it is difficult to measure and evaluate, and the consequences of dependency are also complex [18], which may be positive or negative. Place dependency is usually understood as follows: an individual experiencing a place becomes dependent on the place because the place can fulfill his or her needs, creating dentification, belonging, and other emotional aspects of the performance. Place dependency is a type of functional dependency, reflecting the importance of the resources and facilities provided. A study reported that place dependency is associated with high quality service [19].

Recently, the interest in resource dependency among fields of community psychology [20], environmental psychology [21], and gerontology to explore the provision of community service resources has been increasing [22]. A study has suggested that the survival of individuals or organizations ought to gain resources from their surroundings, and ought to interact with their surroundings to achieve the best survival outcomes [23]. Research has demonstrated that in the past two decades, the number of nursing homes in the world has decreased significantly, and an increasing number of elderly people individuals are living in their homes [24, 25], leading to complex nursing needs for families and community services.

The World Health Organization [26] suggests that the best way to support the elderly people in the community is to integrate all types of resources, adapt to the actual social environment of the elderly people, and provide targeted health services; these integrated services should be prioritized over other services [27]. However, how to achieve or solve the complex problem, including improving the understanding of community needs, remains unclear. In particular, individuals of various ages and levels of social support have various needs for the environment and service resources. As individuals enter old age, increased age introduces another change in social status, economic circumstances, physical function, psychology and cognition, which also has an increased demand for health service resources [28]. Surrounding resources and social support are important determinants of health trajectories and life expectancy in the aging process. Social support had moderating effects between dependency and need for resource-use. High level of social support may reduce dependency through modifying chronic disease status and individual income, while low social support was associated with increased dependent on service resources [29]. According to our review of the literature, the complex association between dependency and the community environment has not been investigated through complete data analysis. Therefore, the present study aims to establish the association between dependency and community resources and clarify the need for resources among elderly people with varying ages and levels of social support, provide evidence for further research into and development of effective strategies to improve the community environment and services on the basis of the needs of the elderly people, and improve community resources.

## Methods

### Study design

This study used a cross-sectional design based on a community-based project that aim to explore accessible health resources to promote the health of the elderly people.

### Setting and participants

**Fig. 1:**
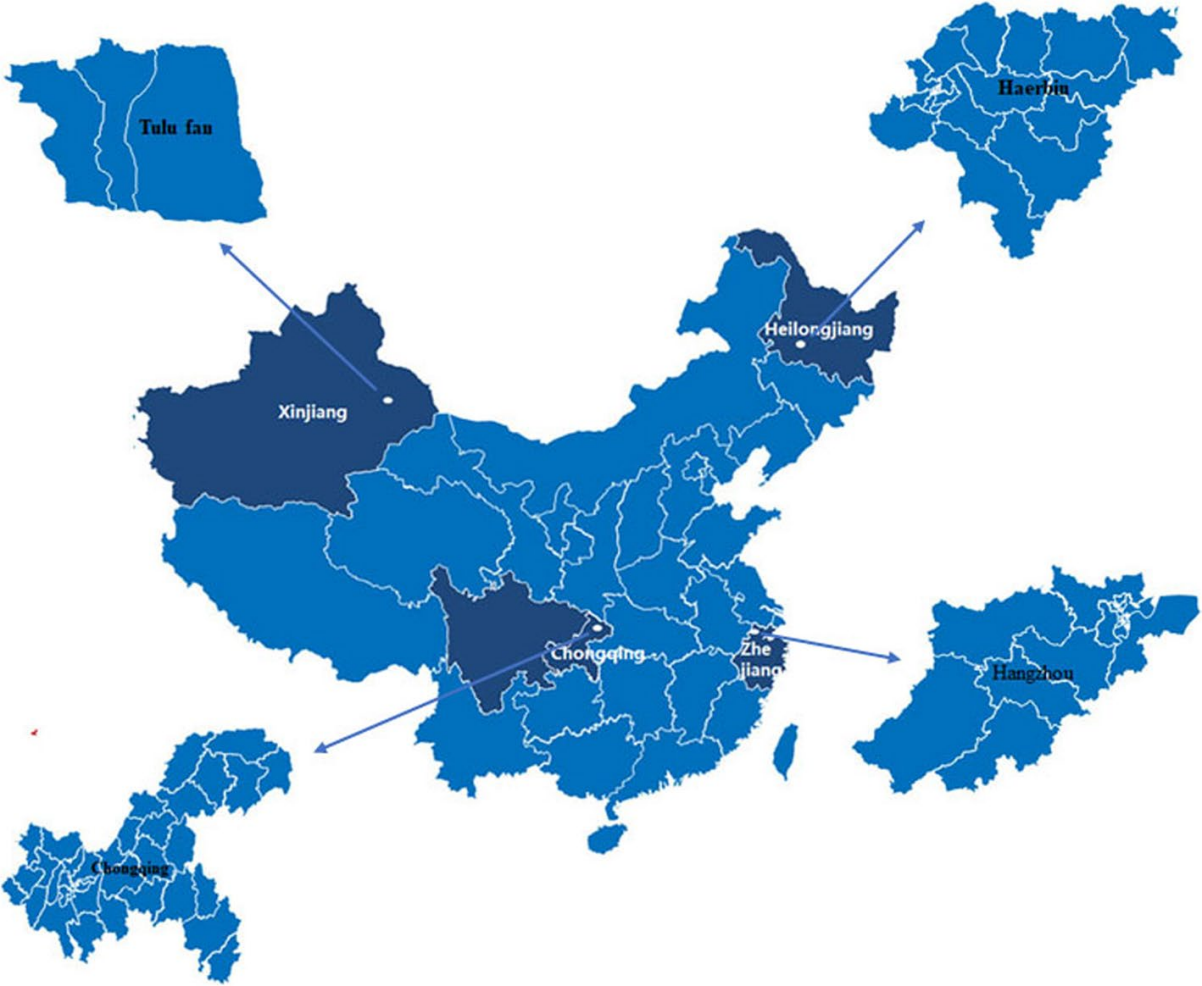
Distribution of study area in China. Resources from: https://www.tocreating.com/ppt/a7kn3.html

A total of 950 elderly people aged 60 years or older participate in the study. The events per variable method is used to determine the sample size for performing an analysis [30]. A multistage sampling design was conducted in China, according to the geographical location and social-economic factors. The secondary sampling cities comprise Chongqing, Harbin, Tulufan and Hangzhou from four sampling provinces (Fig. 1). The third sampling unit included 22 locations from secondary sampling cities. The sample size was different for each sampling unit owing to the difference in sample content and organizational capabilities in the final sampling unit. Eligible participants in accordance with the inclusion criteria are regular inhabitants aged 60 years and above and able to complete the investigation. Individuals will be excluded from this study in accordance with the exclusion criteria, if they have a physical and mental disability. Participants who responded to the questionnaire were recruited by local staff members. Among the participants who responded to the questionnaire, two subjects who were unable to complete the questionnaire and seven subjects aged below 60 years who failed to meet the inclusion criteria were excluded from the study. After 28 subjects with missing data were excluded, there were a total of 913 eligible participants who participated in this study. Written informed consent was obtained from all participants and the study was approved by the local Institutional Review Board.

### Data collection

Data were collected using questionnaires provided to the participants during face-to-face interviews. The nine-part questionnaire comprised 428 response items. Interviews were conducted by researchers, and interviewers with prior interview experience through a university volunteer platform were recruited. The interviewers were trained and conducted preliminary investigation before the formal investigation.

The demographic characteristics and daily habits were reported by participants. Environmental was measured using three dimensions: social, psychological, and physical. In the study, the environmental assessment was determined by assessing the housing environment, surrounding housing environment, and community environment. The environmental resources of community were measured using 44 items [31], and the Cronbach's alpha coefficient (Cronbach's α) was calculated. It is 0.89 for total scale.

The dependency was assessed for every participant using Minnesota Multiphasic Personality Inventory-II. The raw score was calculated by using 57 items, and converted into a T-score [32]. Dependency was defined as a standardized T-score greater than or equal to 60 points [33]. The test–retest reliability was higher in women (0.81) than in man (0.67), and criterion-related validity was -0.70. Older American Resources and Services (OARS) scale was used to assess the social resource of individuals [34, 35]. The scale is comprised of three dimensions: social interaction, availability of social support and practical assistance, and interpersonal relation. The ratings were summed to yield a total score, and Cronbach's α ranged from 0.61 to 0.83 for three scales. Personality were identified using the Eysenck Personality Questionnaire (EPQ) [36]. Depressive symptoms were assessed using the Geriatric Depression Scale. In a previous study, the reliability and validity have been described [37]. We ensure that the instruments were properly used and met reasonable use requirements.

### Statistical analysis

A total of 913 participants were included in the analysis. Dependency scores were calculated and no missing value were accepted. Descriptive analysis was performed to present the general and demographic characteristics of participants.

We conducted cluster analysis for the community environmental variables using the oblique principle component method to analyze the association between dependency and community environmental factors. Univariate analyses were performed to determine whether the variable was added into the cluster analysis model with a power of 80% at a significance level of 0.05. The R-squared with own cluster and R-squared with 1-R^2^ ratio were used to determine the number of clusters. The cluster trees is output by the program “proc tree.”

The logistic regression analysis was performed to assess the association between dependency and community environment resources and the associated risk factors. The dependency score was treated as a binary variable. A cut-off points was determined for dependency. Standard T-score is 60 points for the cut-off value. The model included all the community environment variables that were significant in the univariate analysis, adjusted for important confounding factors such as demographic variables, daily behavior habits and psychological variables, and social support level.

We conducted two separate logistic regression analyses by the age and levels of social support, to compare the association between dependency and community environment resources in different age groups and levels of social support. The age was divided into two categories: under 70 years and over or equal to 70 years. The levels of social support were divided into two categories: less than 11 points and greater or equal to 11 points by the median. Models were also adjusted for the level of physical activity, chronic disease status, gender, income satisfaction, EPQ scores, alcohol consumption, and smoking status. All analyses were performed using SAS for Windows (version 9.4).

## Results

Table 1 showed the general characteristics of the study participants, and the highlights are as follows: 37.7% were aged over 70 years, 58.4% were women, 41.6% were men, 19.6% were non-married, and 66.7% had been diagnosed with chronic diseases. 12.8% of the participants were assessed as having dependency, and no significant difference between women and men was observed.

**Table 1:** Characteristics of study participants in the study

Variable categories	n	%
Characteristic variables (n, %)		
Sex		
Male	380	41.6
Female	533	58.4
Marital status		
Married	734	80.4
Non-married	179	19.6
Education levels (yr)		
0–6	176	19.3
7–9	353	38.7
10–12	211	23.1
13 +	173	18.9
Individual income		
¥0 to 1,999	550	60.2
¥2,000 to 3,999	230	25.2
¥4,000 to 5,999	87	9.5
¥6,000 and Over	46	5.1
Smoking status		
Yes	126	13.8
No	787	86.2
Alcohol use		
Yes	186	20.4
No	727	79.6
Physical activity		
Yes	360	39.4
No	553	60.6
Chronic disease status		
Yes	611	66.9
No	302	33.1
Measured Variables (Mean, SD)		
Age (yr)	68.6	6.5
Dependency scores	43.4	12.8
GDS-15 scores	3.4	3.0
EPQ scores	22.3	4.1

*GDS-15* The 15-item Geriatric Depression Scale

*EPQ* The Eysenck Personality Questionnaire

**Fig. 2:**
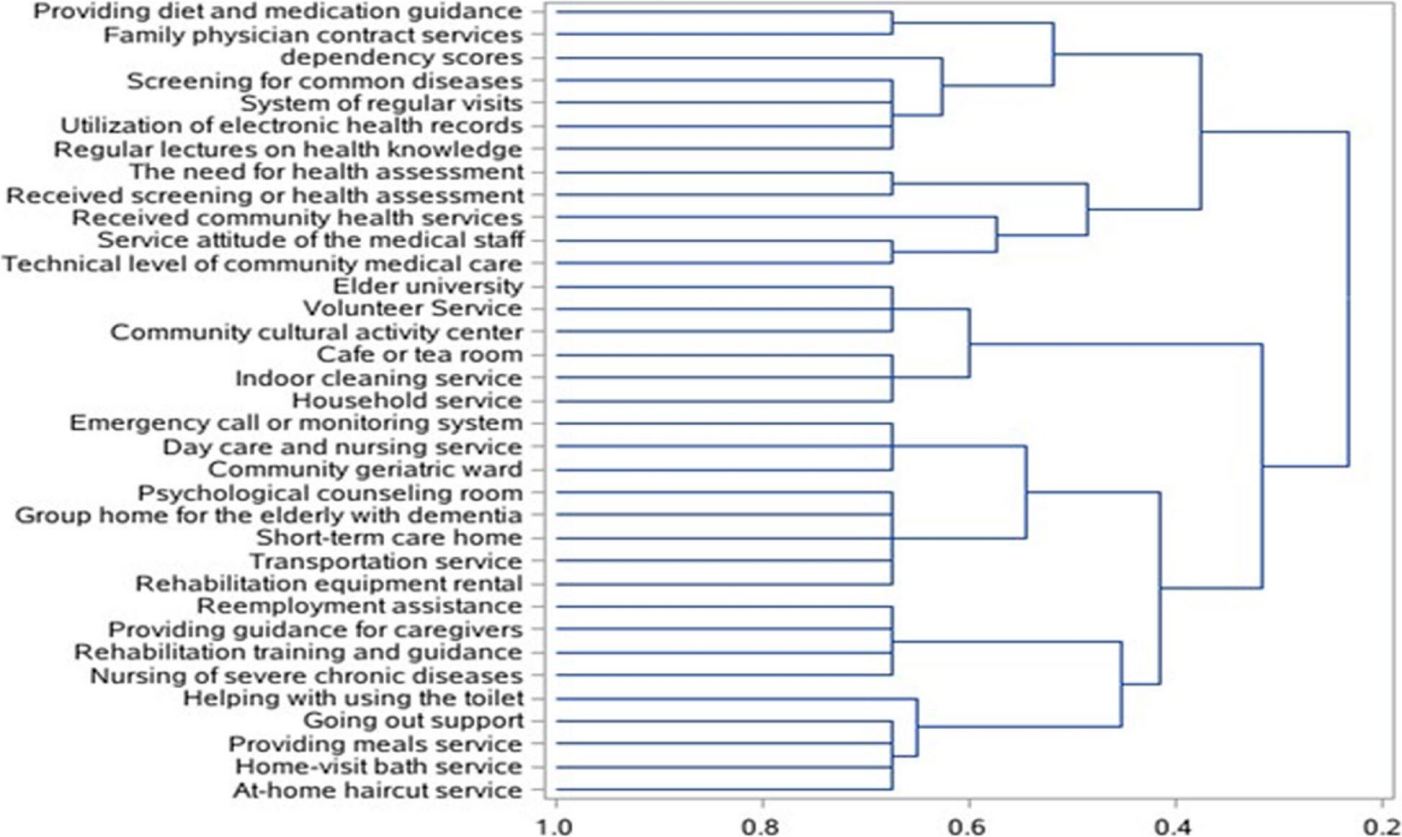
The tree graph of community environment resources by cluster analysis

The tree graph by cluster analysis is presented in Fig. 2. The 34 variables of the community environment that were significantly associated with dependency were divided into six categories. The first category can be summarized as follows: community primary preventive care service resources (e.g., diet and health guidance for elderly people and chronic patients), contracted family doctor services, screening for common diseases, regular home visits for the elderly people living alone and disabled, the utilization of electronic health records, and holding regular lectures on general health knowledge. Other community environment variables can be classified into the following categories: utilization of and satisfaction with community services, community culture, and basic living facilities; psychological, cognitive, and comprehensive nursing resources; employment and caregiver guidance organizations; and home living support services. We put the score of dependency into the cluster analysis model to investigate the association between the score of dependency and the environmental resources of the community. The score of dependency was classified into the category of community primary preventive care service resources, and no significant change in those variables in another category was observed.

Table 2 presents the odds ratios (ORs) of the community environment resources for dependency using logistic regression models. The participants who responded “yes” to “Do you think it is necessary for community medical staff to assess your health status” and “if there is a short-term care home for the elderly people in your community” were compared with the participants who responded “no” to the same items. The result was as follows: these community environment variables were significantly positively associated with levels of dependency in the logistic regression analysis, namely the OR was 2.10 (95% CI, 1.33–3.34, *P* = 0.001) and 2.40 (95% CI, 1.40–4.09, *P* = 0.001). Other community environmental resources such as the community geriatric ward, regular lectures on health knowledge, and screening for common diseases were also positively associated with levels of dependency: the OR was 1.97 (95% CI, 1.23–3.18, *P* = 0.005), 1.50 (95% CI, 1.06–2.14, *P* = 0.023), and 1.56 (95% CI, 1.05–2.34, *P* = 0.029), respectively. The community cultural activity center, supermarket or agricultural market, and volunteer service were negatively associated with levels of dependency: the OR was 0.50 (95% CI, 0.34–0.69, *P* < 0.001), 0.57 (95% CI, 0.38–0.86, *P* = 0.007), and 0.56 (95% CI, 0.40–0.79, *P* = 0.001), respectively.

**Table 2:** The odds ratios of community environment resources and other related risk factors for dependency by logistic regression models

Variables	Multivariable adjusted Odds Ratios			
		95% CI		*P* value
Received community health services (times)	1.27	1.08	1.48	0.004
The need for health assessment (n/y)	2.10	1.33	3.34	0.001
Community geriatric ward (n/y)	1.97	1.23	3.18	0.005
Short-term care home (n/y)	2.40	1.40	4.09	0.001
Regular lectures on health knowledge (n/y)	1.50	1.06	2.14	0.023
Screening for common diseases (n/y)	1.56	1.05	2.34	0.029
Community cultural activity center (n/y)	0.49	0.34	0.69	< 0.001
Volunteer Service (n/y)	0.57	0.38	0.86	0.007
Supermarket and agricultural market (n/y)	0.56	0.40	0.79	0.001
Age (y)	1.03	1.01	1.06	0.004
Social support (score)	0.86	0.82	0.91	< 0.001
Chronic disease status (y/n)	0.71	0.52	0.96	0.027
Income satisfaction (n/y)	1.61	1.39	1.87	< 0.001
EPQ scores (points)	1.13	1.09	1.17	< 0.001

The age-specific results by separated logistic regression models are shown in Table 3. In the group aged under 70 years, the utilization of electronic health records and the need for health assessments, rehabilitation equipment rentals, and community nursing homes were significantly associated with the levels of dependency scores: the OR was 2.81 (95% CI, 1.90–4.14, *P* < 0.0001), 2.25 (95% CI, 1.24–4.06, *P* = 0.007) and 2.13 (95% CI, 1.02–4.43, *P* = 0.043), and 1.90 (95% CI, 1.12–3.24, *P* = 0.018), respectively. The community cultural activity center and supermarket or agricultural market were negatively associated with levels of dependency.

**Table 3:** The odds ratios of community environment resources for dependency by the age-specific

	Variables	Multivariable adjusted Odds Ratios	95% CI		*P* value
Age < 70	Received community health services	1.26	1.03	1.55	0.024
	Utilization of electronic health records	2.81	1.90	4.14	< 0.001
	The need for health assessment	2.25	1.24	4.06	0.007
	Rehabilitation equipment rental	2.13	1.02	4.43	0.043
	Community nursing home	1.90	1.12	3.24	0.018
	Community cultural activity center	0.35	0.22	0.56	< 0.001
	Supermarket and agricultural market	0.57	0.38	0.85	0.006
Age ≥ 70	Received community health services	1.49	1.18	1.88	< 0.001
	Short-term care home	4.01	1.64	9.80	0.002
	Day care and nursing service	2.41	1.13	5.17	0.024
	Transportation service	1.86	1.03	3.36	0.040
	Regular lectures on health knowledge	1.93	1.21	3.08	0.006
	Canteens for the elderly	0.30	0.13	0.71	0.006
	Volunteer Service	0.38	0.21	0.70	0.002
	Cafe or tea room	0.50	0.26	0.95	0.034
	Library	0.46	0.24	0.87	0.018

In the group aged 70 years and over, a short-term care home was strongly associated with levels of dependency: the OR was 4.01 (95% CI, 1.64–9.80, *P* = 0.002). The daycare and nursing service, transportation service, and regular lectures on health knowledge were associated with levels of dependency: the OR was 2.41 (95% CI, 1.13–5.17, *P* = 0.024), 1.86 (95% CI, 1.03–3.36, *P* = 0.040), and 1.93 (95% CI, 1.21–3.08, *P* = 0.006), respectively. The canteens for the elderly people, volunteer service, libraries, and cafes or tea rooms were negatively associated with levels of dependency. The received community health services were positively associated with levels of dependency in the groups aged under as well as over 70 years.

The social support levels-stratified results using the separated logistic regression models are shown in Table 4. The number of times that community health services were received was significantly associated with levels of dependency in both the high level of social support group and the low level of social support group. In the group with a low level of social support, the following were significantly associated with levels of dependency: an emergency call or monitoring system, transportation services, the need for health assessment, and regular lectures on health knowledge: the OR was 2.42 (95% CI, 1.29–4.52, *P* = 0.006), 2.19 (95% CI, 1.18–4.07, *P* = 0.013), 1.89 (95% CI, 1.06–3.36, *P* = 0.031), and 1.98 (95% CI, 1.29–3.02, *P* = 0.002), respectively. The volunteer service and cafe or tea room were negatively associated with levels of dependency.

**Table 4:** The odds ratios of community environment resources for dependency by the social support levels-specific

	Variables	Multivariable adjusted Odds Ratios	95% CI		*P* value
LSS^*^	Received community health services	1.28	1.02	1.60	0.036
	Emergency call or monitoring system	2.42	1.29	4.52	0.006
	Transportation service	2.19	1.18	4.07	0.013
	The need for health assessment	1.89	1.06	3.36	0.031
	Regular lectures on health knowledge	1.98	1.29	3.02	0.002
	Volunteer Service	0.44	0.25	0.76	0.004
	Cafe or tea room	0.30	0.17	0.51	< 0.001
HSS^*^	Received community health services	1.54	1.25	1.89	< 0.001
	Community geriatric ward	2.37	1.24	4.53	0.009
	Short-term care home	2.93	1.37	6.25	0.006
	Regular lectures on health knowledge	1.82	1.15	2.89	0.011
	System of regular visits	1.95	1.25	3.06	0.004
	Emergency call or monitoring system	0.40	0.21	0.77	0.006
	Elder university	0.50	0.30	0.86	0.011
	Supermarket and agricultural market	0.52	0.33	0.80	0.003

^*^LSS Low levels of social support

^*^HSS High levels of social support

In the group with a high level of social support, community geriatric ward, short-term care home, regular lectures on health knowledge, and regular home visits for the elderly people who were living alone and disabled were associated with the levels of dependency: the OR was 2.37 (95% CI, 1.24–4.53, *P* = 0.009), 2.93 (95% CI, 1.37–6.25, *P* = 0.006), 1.82 (95% CI, 1.15–2.89, *P* = 0.011), and 1.95 (95% CI, 1.25–3.06, *P* = 0.004), respectively. An emergency call or monitoring system, elder universities, and a supermarket or agricultural market were negatively associated with levels of dependency.

## Discussion

In the present study, we drew from resource dependency theory to explore the association between dependency and community environmental resources. Our results showed that community environment resources were significantly associated with dependency among elderly people. The association between dependency and community environmental resources differed among elderly people by age group and social support level.

The incidence of dependency steadily increases because of population aging, chronic health conditions, and changes in society [38]. In traditional countries, the family is the main healthcare resource. However, with the change in the world’s population structure and social economy, the family is no longer the main healthcare resource [39, 40]. Studies have shown that dependency can lead to prolonged hospital stays, increases in unnecessary medical costs, and excessive dependence on caregivers, all of which adversely affect the physical and mental health of elderly people [41]. Although these studies have revealed that dependency can lead to adverse outcomes and consequences, they have not yet explored the improvement strategy by considering the dependency object as the resource demand. The increasing demand from elderly people for complex care requires sustained input from family caregivers or community health or social services to support independent living [42]. Even in resource-rich countries, the demand for home care services has increased. Alternative services have been developed to enable individuals to remain independent in terms of personal care activities if possible or to be provided with such care activities through the improvement of services. Such services may enhance independence, reduce dependency, and lead to cost reductions [43, 44]. At present, research focuses more on what communities can do to promote health equity as well as what methods or ways are expected to support them through many stakeholders [45].

In addition, elderly people are usually considered a vulnerable group that is likely to be affected by a poor community environment. A number of studies have reported that the elderly people group spends a lot of time in the community and is more dependent on local resources and services compared to other groups [46]. Community environments may play a major role in supporting healthy aging, and the provision of friendly living environments for elderly people is a public health concern that should be on the agenda of local governments [47]. However, an evidence-based approach is necessary to understand the interactions between community environmental and health demands among elderly people. Therefore, based on the theory of resource dependency, this study used the logistic regression model to quantitatively evaluate the association between community environmental resources and dependency. This analysis showed that a resource-rich community environment was associated with the reduced risk of dependency among elderly people. Resources for elderly people, such as community cultural activity centers, libraries, cafes or tea rooms, universities, supermarkets and agricultural markets, canteens, volunteer services, and emergency call or monitoring systems, were negatively associated with dependency.

Several studies have assessed the association between community environment and the health of elderly people who reside in it, but they focused more on behaviors, satisfaction with living, and depression [48]. However, the measurements of community environment considerably vary between research fields. The U.S. Department of Housing and Urban Development developed the Healthy Communities Assessment Tool to assess the community environment from the following aspects: physical, social, and economic roots of community health [49]. The Public Health Agency of Canada established the Age-Friendly Communities Evaluation Guide to evaluate their age-friendly community initiatives [50]. Age-friendly communities help their older residents remain healthy, active, and independent and enable them to make important contributions as they age. The measurable indicators applicable to the eight domains of community life include the 43 items being addressed in age-friendly programming. Studies have shown that physical, social, and psychological environments may affect an individual’s mental health and have suggested that the environment may play a particularly important role in the mental health of older adults compared with younger adults. However, studies focusing on dependency and the community environment have paid little attention to other environmental factors or have weak research designs, often without control for confounding variables. In this study, we used the EPQ scale to assess the personality characteristics of the participants, and the association between community environmental factors and dependency was adjusted using the EPQ score to remove the effect of the important risk factor. Notably, studies have not comprehensively explored this association.

Studies have shown that a high level of social support may reduce the dependency of elderly people [51]. We divided the participants into two groups according to their social support score. In the group with a low level of social support, the emergency call monitoring system was significantly associated with a high level of dependency. The common characteristics of elderly people with a low level of social support are that they live separate from their families, have fewer social interactions and outings, have a sense of loneliness, and are often left unattended when ill. Therefore, they must be provided with a system or service that makes them feel safe and secure in their own homes [52, 53]. For elderly people with a high level of social support, dependency was associated with short-term care homes [54, 55]. This group prefers to live at home with their families and only uses short-term care temporarily when they are sick or need treatment. In the present study, we addressed the community resources of dependency related for elderly people of varying ages. Elderly people under the age of 70 are dependent on primary healthcare resources such as health assessment and electronic health records because their physical function and internal ability are in a relatively stable stage. Those elderly people with rehabilitation equipment rental were associated with high levels of dependency. Meanwhile, elderly people over 70 years old are more dependent on short-term care home, day care and nursing, and transportation services owing to the gradual decline of their physical and overall functions. Volunteer service may help reduce their dependence on health service resources. These findings can be used for reference as a priority development project of the community health service system.

Few studies have discussed the association between dependency and community environmental resources from the perspective of resource dependency. One study demonstrated that the understanding of dependency on community services usually has negative characteristics [56]. However, dependency is not a totally negative or positive concept; instead, a gradient exists between negative and positive understandings. Dependency on health services can be described as a positive condition for patients because it helps them feel as if they are in a safe and comfortable place. More recently, dependency on community services has been regarded as being on a continuum of more or less autonomy. The situation may also be an often necessary and helpful temporary phase toward full autonomy. It is the transitory and ever-changing state of dependency on community services that contrasts with a static form of institutional dependency and makes dependency not an entirely negative phenomenon.

With aging, elderly people become more vulnerable to environmental challenges. Additionally, dependency and depression increase with age and are mainly caused by social factors, mostly in vulnerable groups and elderly people with a low social support level. The community environment is particularly important for the well-being of elderly people, especially for their mental health [57, 58]. A study suggests that a large number of potential benefits can be attributed to community participation. It is better approach to address the needs of community through community management processes and promoting health [59]. Therefore, based on the resource dependency theory, our study’s discussion focused on the demand for resources. We used quantitative analysis to evaluate the association between dependency and community environmental resources. These results also verified the association between community environmental resources and the mental health of elderly people and provided a scientific basis for improving community resources and dependency intervention.

Several limitations in this study need to be addressed. First, the association between dependency and community environmental was based on a cross-sectional design and so the causality could not be discussed. The complex and changing trend of environmental factors and the change in the association of dependency over time could not be evaluated and observed. For example, whether the increased allocation of elderly-friendly facilities in the community (e.g., community cultural activity centers, elder universities, and cafes or tea rooms) helped elderly people reduce their utilization of and dependency on community primary preventive care service resources was not confirmed by our study design. In addition, although our study fully considered the variables of community environmental resources and the important risk factors, our sample size was too small to evaluate the interactions between the community environment and dependency. Thus, further research is necessary to explore the strategies and effects of improving community environmental dependence.

## Conclusions

This study applied the theory of resource dependence to examine whether the community environment was significantly associated with dependency among elderly people. The community primary preventive care service resources were associated with increased levels of dependency, and the community cultural and living facilities were associated with reduced levels of dependency among the elderly people in the community. Our results suggest that dependency on community environment resources leads to a combination of negative and positive experiences. Additionally, our results might guide future evaluations of community resources provided by such services, to improve the understanding of what interventions must be implemented to further support the elderly people in the community in their lives and promote their autonomy. Through improving the environment, a positive community for the elderly people can be created, enabling them to extend the amount of time they can remain in their residence.

## Supplementary Information

**Additional file 1. **Questionnaire.

## Data Availability

The datasets generated and analysed during the current study are not publicly available due to ensure the privacy of participants but are available from the corresponding author on reasonable request. Source of map are available from: https://www.tocreating.com/ppt/a7kn3.html, we received permission to use and adapt the figures.

## Abbreviations

Cronbach's α: Cronbach's alpha coefficient
EPQ: Eysenck Personality Questionnaire
OARS: Older American Resources and Services
ORs: Odds Ratios
